# Foot Conditions among Homeless Persons: A Systematic Review

**DOI:** 10.1371/journal.pone.0167463

**Published:** 2016-12-09

**Authors:** Matthew J. To, Thomas D. Brothers, Colin Van Zoost

**Affiliations:** 1 Faculty of Medicine, Dalhousie University, Halifax, Nova Scotia, Canada; 2 Walk in Our Shoes Foot Care Association, Halifax, Nova Scotia, Canada; Leibniz Institute for Prvention Research and Epidemiology BIPS, GERMANY

## Abstract

**Introduction:**

Foot problems are common among homeless persons, but are often overlooked. The objectives of this systematic review are to summarize what is known about foot conditions and associated interventions among homeless persons.

**Methods:**

A literature search was conducted on MEDLINE (1966–2016), EMBASE (1947–2016), and CINAHL (1982–2016) and complemented by manual searches of reference lists. Articles that described foot conditions in homeless persons or associated interventions were included. Data were independently extracted on: general study characteristics; participants; foot assessment methods; foot conditions and associated interventions; study findings; quality score assessed using the Downs and Black checklist.

**Results:**

Of 333 articles screened, 17 articles met criteria and were included in the study. Prevalence of any foot problem ranged from 9% to 65% across study populations. Common foot-related concerns were corns and calluses, nail pathologies, and infections. Foot pathologies related to chronic diseases such as diabetes were identified. Compared to housed individuals across studies, homeless individuals were more likely to have foot problems including tinea pedis, foot pain, functional limitations with walking, and improperly-fitting shoes.

**Discussion:**

Foot conditions were highly prevalent among homeless individuals with up to two thirds reporting a foot health concern, approximately one quarter of individuals visiting a health professional, and one fifth of individuals requiring further follow-up due to the severity of their condition. Homeless individuals often had inadequate foot hygiene practices and improperly-fitting shoes. These findings have service provision and public health implications, highlighting the need for evidence-based interventions to improve foot health in this population. An effective interventional approach could include optimization of foot hygiene and footwear, provision of comprehensive medical treatment, and addressing social factors that lead to increased risk of foot problems. Targeted efforts to screen for and treat foot problems could result in improved health and social outcomes for homeless individuals.

## Introduction

Homelessness is a major public health concern in North America. Recent reports suggest that on any given night, over 700,000 individuals across the United States and Canada are homeless. [1], [2] Homeless individuals have significantly higher rates of mortality, morbidity, and hospitalization compared to the general population. [3] They face a wide range of health problems such as seizures, mental illnesses, respiratory diseases, and dental problems, but frequently report unmet needs for health care. [3], [4]

Foot problems have been described as a common concern among homeless individuals, but these are often overlooked and inadequately treated. [3], [5], [6] Walking is a common mode of transportation among homeless individuals and increased risks of physical injury, poor hygiene, and inadequate footwear have been cited as contributing factors to the development of foot problems. [6] Lack of access to health services and financial resources also prevent homeless individuals from receiving appropriate treatment for foot-related concerns. [6] Although a small number of studies have been conducted, the foot health concerns of homeless individuals have not been systematically investigated.

Given that foot problems are common among homeless persons, it is important to summarize the evidence on this topic to inform patient care and public health approaches. The objectives of this systematic review were to summarize current published literature related to foot conditions and associated interventions among homeless persons.

## Methods

### Overview

The review was guided by methodology outlined by the PRISMA statement (S1 Checklist). [7] A systematic search strategy was developed to identify articles that reported foot problems among homeless populations. A protocol of the review has not been previously published.

### Eligibility Criteria

All original research articles that reported on foot conditions in homeless persons were included in the review. Articles that did not present data on clear foot health outcomes (e.g. reviews, case reports, and commentaries) were excluded.

### Information Sources and Search Strategy

Articles were identified through a search of MEDLINE (1966–2016), EMBASE (1947–2016), and CINAHL (1982–2016) with no language exclusions. The search strategy was developed by one member of the research team (MJT) in consultation with a health sciences librarian and included terms related to homelessness that were cross-matched with foot health terms (Box 1). The yield from bibliographic databases was complemented by manual searches of reference lists from relevant research articles. The search concluded in July 2016.

**Box 1. MEDLINE, EMBASE, and CINAHL search strategies**

**MEDLINE**

(“Homeless Persons” [MeSH] OR “Homeless Youth” [MeSH] OR homeless*[all fields] OR fixed address[tw] OR runaway* OR street person[tw] OR street people[tw] OR street youth*[tw] OR squatter* OR underhouse* OR roofless* OR seeking shelter[tw] OR shelter seeking[tw] OR unhouse* OR street involved[tw] OR sleeping rough[tw] OR unstable hous*[tw] OR unstably hous*[tw] OR housing instability[tw] OR precarious hous*[tw] OR precariously hous*[tw] OR vulnerable hous*[tw] OR vulnerably hous*[tw] OR "Vulnerable Populations"[Mesh]) AND (“Podiatry” [mh] OR Podiatr* OR chiropod* OR foot* OR feet* OR “Foot Diseases” [MeSH] OR “Onychomycosis” [MeSH] OR onychomycos* OR “Diabetic Foot” [mh] OR diabetic feet[tw] OR “Tinea Pedis” [mh] OR athlete's foot[tw])

**EMBASE**

(‘homelessness’/exp OR homeless* OR ‘no fixed address’ OR runaway* OR ‘street person’ OR ‘street persons’ OR ‘street people’ OR ‘street youth’ OR ‘street youths’ OR squatter* OR underhouse* OR roofless* OR seeking NEAR/3 shelter OR unhouse* OR ‘street involved’ OR sleeping NEAR/3 rough OR unstabl* NEAR/3 hous* OR ‘housing instability’ OR precarious* NEAR/3 hous* OR vulnerabl* NEAR/3 hous*) AND (‘podiatry’/exp OR podiatr* OR chiropod* OR foot* OR feet* OR ‘foot disease’/exp OR ‘onychomycosis’/exp OR onychomycos* OR ‘diabetic foot’/exp OR ‘diabetic feet’ OR ‘tinea pedis’/exp OR athletes foot)

**CINAHL**

(MH “Homeless Persons” OR MH “Homelessness” OR TX homeless* OR TX “fixed address” OR TX runaway* OR TX “street person*” OR TX “street people*” OR TX “street youth*” OR TX “squatter*” OR TX “underhouse*” OR TX “roofless*” OR seeking N3 shelter OR TX unhouse* OR street N3 involved OR sleeping N3 rough OR TX hous* N3 instability OR TX hous* N3 (unstable OR unstably) OR TX hous* N3 (precarious OR precariously) OR TX hous* N3 (vulnerable OR vulnerably) OR MH “Special Populations”) AND (MH “Podiatry” OR MH “Podiatry Practice” OR Podiatr* OR chiropod* OR MH “Podiatric Care” OR MH “Foot Care” OR MH “Foot Diseases” OR MH “Onychomycosis” OR TX onychomycos* OR MH “Diabetic Foot” OR TX "diabetic feet*" OR TX “Tinea Pedis” OR TX “athlete's foot”)

### Study Selection

Titles and abstracts of potentially eligible articles were screened by two independent reviewers (MJT, TDB). If it was unclear whether an article met the inclusion criteria, the full-text article was retrieved to determine eligibility. Articles that met the inclusion criteria were included in full-text analysis. Discrepancies in the screening process were resolved by consensus.

### Data Extraction and Analysis

A standardized data extraction form was developed by the research team. Data were extracted by two independent reviewers (MJT, TDB) on the following variables: general study characteristics (e.g. primary author, year of publication, country of study, and source of funding); participants (age, sex, and sociodemographic data); study design; foot assessment methods; foot conditions and associated interventions; study findings. Quality of reports was independently assessed by two research team members (MJT, TDB) using the Downs and Black checklist. [8] The checklist has high internal consistency (Kruder-Richardson formula 20 = 0.89), test-retest reliability (r = 0.88), and inter-rater reliability (r = 0.75). The 27-item checklist assesses items related to reporting (clear description of objectives, participants, outcomes, findings, confounders, loss to follow-up, probability values), internal validity (biases, statistical analyses), external validity (representativeness of participants and treatment setting), and power. For the current review, 6 items related to randomized trials were excluded as no randomized interventions were identified. The checklist item that assessed adequacy of a study’s statistical power was simplified to a score of 0 or 1. The maximum quality score that could be attained was 22. Discrepancies were resolved by consensus. Data on all study variables were summarized. Quantitative variables were analyzed using Microsoft Excel.

## Results

**Fig 1:**
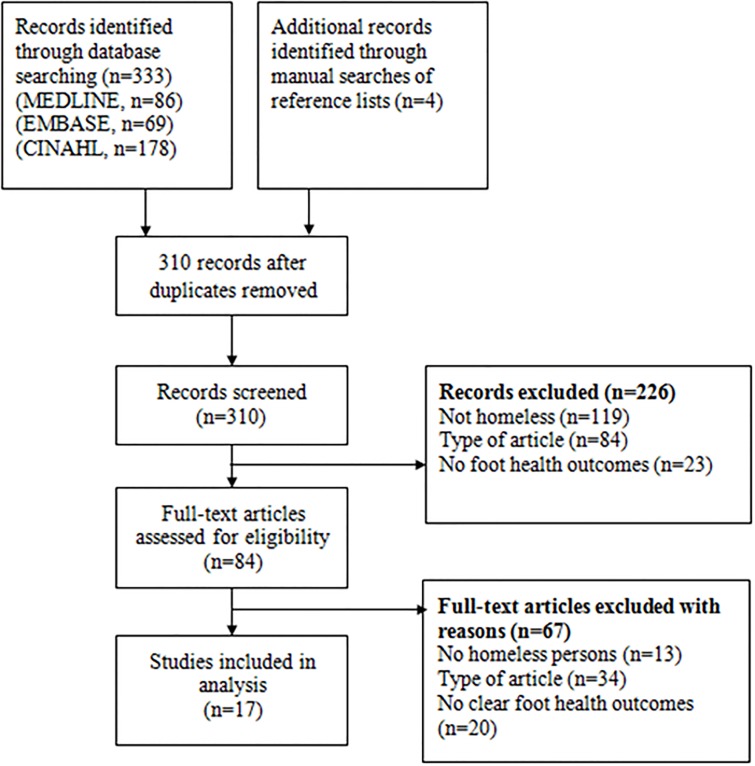
Flow diagram of articles identified, screened, and included in analysis.

The search yielded potentially eligible articles. After screening their titles and abstracts, 81 articles were deemed potentially relevant and full texts were retrieved to determine eligibility. Of these, 64 were excluded because they did not include homeless persons, did not present original research, or did not include data on clear foot health outcomes (S1 Box). Therefore, 17 articles were selected for inclusion in the analysis (Fig 1). Characteristics of included studies are presented in Table 1.

**Table 1:** Characteristics of 17 included studies.

First author (year)	Sample size (% male); % homeless	Mean age (range)	Setting	Assessment of foot conditions	Study findings	Quality score^1^
Matteoli(2015)	930 (87%); 100%	43 (SD = 12); 24% were between 30–35 years old	Volunteer association that provided health services and outreach in 3 municipalities of Rome, Italy	Questionnaire, clinical exam, diabetic foot intervention	Of 930 homeless participants, 21 (2.2%) had diabetic foot ulcers and received a total of 369 procedures. A mean of 18 +/- 14 interventions were necessary to resolve foot problems. Diabetic ulcers were treated weekly with regular medication for a mean SD of 17.6 +/- 12 months. Significant clinical improvement was observed in 18 patients (86%).	15
Chong (2014)	95(74%); 100%	48 (20–72)	2 homeless shelters in Long Beach, CA, USA	Questionnaire, medication review	37% reported foot problems. There were no significant differences in the reporting of foot problems between homeless men and women.	17
Chen (2014)	299 (92%); 100%	NR; 62% were between 36–55 years old	2 homeless shelters in San Francisco, CA, USA	Questionnaire	In the sample of 299 participants, common foot problems were foot pain (56%), fungal nail (30%), prior foot injuries (27%), calluses (26%), athlete’s foot (24%), and corns (19%). Other conditions included ingrown nails (15%), bunion (14%), hammertoe (7%), gout (6%), immersion foot (5%), ulcers (4%), warts (4%), peripheral artery disease (3%), diabetes mellitus type 2 (5%), diabetes mellitus type 1 (4%), and frostbite (2%).	14
Schwarzkopf (2011)	235 (71%); 53%	46.5 (18–82)	3 foot clinics in New York City, USA	Retrospective chart review, clinical exam	43.5% of homeless patients had foot-shoe size mismatch of greater than 1 size and 16.9% had mismatch >1.5 size, 6.5% of homeless clinic users had diabetes. Homeless individuals had a significantly higher rate of foot-shoe size mismatch compared to other clinic patients.	11
Muirhead (2011)	100 (65%); 100%	43.2 (SD = 10.9)	Community kitchen in Tennessee, USA	Questionnaire	56% of participants had diabetes, hypertension, peripheral vascular disease, or a combination of the three. 92% valued healthy feet and education related to foot care. 62% felt the condition of their feet was a deterrent to accessing foot care.	10
Arnaud (2009)	488 (80%);^2^ 100%	Diabetics: 53.5 (34–73); non-diabetics: 45.4 (18–85)	9 homeless shelters in Paris, France	Questionnaire, clinical exam	Screening of participants identified 35 previously diagnosed and 2 newly diagnosed individuals with diabetes. Estimated prevalence of diabetes was 6.2%. 41% of diabetic patients had difficulty walking, 42% had a loss of foot sensitivity, and 17% had had a lower limb amputation. 1 in 3 homeless persons with diabetes had high podiatric risk.	16
Schanzer (2007)	445 (51%); 100%	36.9 (18–65)	Homeless shelter in New York City, USA	Questionnaire	12.4% of participants reported podiatric complaints at baseline which decreased significantly to 5.7% who reported podiatric complaints at 18 months after baseline.	17
Badiaga (2005)	698 (94%); 71%	Homeless: 41 (SD = 14.6); Control: 35.4 (SD = 12.6)	2 homeless shelters and travel clinic in Marseilles, France	Case control study, clinical exam	Homeless individuals were more likely to have tinea pedis and scratching lesions of socks compared to controls.	12
Gelberg (2000)	363 (80%); 100%	38.2 (18–70)	Clinics in Los Angeles, USA	Interview, clinical exam	36% of homeless adults had foot, leg, or skin problems based on interview or clinical exam. Foot, leg, or skin conditions were the most common reason for referral.	20
Stratigos (1999)	142 (100%); 100%	38.9 (27–50.8)	Homeless shelter in Boston, USA	Questionnaire and clinical exam	Most prevalent skin diseases in population were tinea pedis (38%), pitted keratolysis of the feet (20.4%), toenail onychomycosis (15.5%), calluses (7.7%)	10
Kleinman (1996)	363 (70%); 100%	37.6 (18–70)	Shelters, meal facilities, and streets in Los Angeles, USA	Clinical exam	24% had self-reported foot abnormalities while 18% had foot abnormalities upon clinical exam. Referrals were indicated in 3/15 foot problems.	14
Macnee (1996)	214 (62%); NR	NR (13–79)	Screening clinics in Johnson City, TN, USA	Chart review, clinical exam	Skin problems, loss of sensation, ill-fitting shoes, diabetes, and poor circulation were identified at foot screening clinic. Of 58 homeless individuals who presented to diabetes screening clinic, 7 (12%) patients had foot problems.	11
Robbins (1996)	461 (53%); 100%	38.6 (11–74)^3^	Screening clinic in Cleveland, USA	Questionnaire, clinical exam	Over 2 years, common foot problems were nail pathology (63–65%), corn and calluses (53–57%), fungal disease (33–53%), neurologic (37–43%), foot injury (24–43%), and bunions (33–43%). Foot problems related to diabetes, flat feet, and plantar warts were also noted.	8
Jones (1990)	511 (83%); 100%	Women: 37.8 (23–77); men: 43.7 (21–75)	5 homeless shelters in Chicago, USA	Chart review	Calluses and corns, dystrophic nails, tinea pedis, ingrown toenails were common among participants. Other findings included macerated skin, blisters, fractures, pain, trauma, frostbite, cellulitis, gout, secondary syphilis	12
Gelberg (1990)	464 (56%); 46%	32 (18–78)	Medical center in Los Angeles, CA	Interview, clinical exam	Homeless participants were more likely to report pain in their feet and functional limitations when walking compared to housed participants.	16
Toon (1987)	266 (95%); 82%	NR; men: mode 40–45; women: 8 were < 35 years old.	Medical clinic in London, UK	Audit of chiropody cases	58% of patients were rated to have good foot hygiene. 21.1% had severe foot problems such as ulceration and blistering. 14% had foot deformities.	12
MacIntyre (1979)	297 (83%); 82%	NR; 70% were between 35–65 years old	Clinic in Glasgow, UK	Chart review	62 (21%) presented to clinic with foot problems. 9 required outpatient hospital referral and 4 required in patient referral.	11

Abbreviations: SD = standard deviation; NR = not reported

^1^Assessed by Downs and Black checklist

^2^Value shown is for diabetic patients. For non-diabetic patients, % male was 81%.

^3^Values shown are for study sample in 1994. In 1995 sample, mean was 37.5 (1–76).

### Setting

Twelve studies included in the review were conducted in the United States, [9]-[20] two in the United Kingdom, [21], [22] two in France, [23], [24] and one in Italy. [25] The majority of studies included homeless shelter residents or patients who attended clinics dedicated to serving vulnerable populations. Seven (50%) studies included patients from clinics that served homeless individuals, [9], [10], [13]-[15], [21], [22] while eight (47%) studies recruited participants directly from homeless shelters. [11], [12], [16], [17], [19], [20], [23], [24] One study recruited homeless individuals from a volunteer health service outreach association, [25] and another study recruited homeless individuals from a community kitchen. [18] Ten (59%) studies included participants from more than one location (e.g. shelters, clinics). [10], [11], [13], [15], [16], [19], [29], [23]-[25]

### Sample

Studies included a median number of 363 participants (range 95–930). The mean age of participants across studies was 40 years old (mean ages ranged from 32–48 years old). Five studies did not provide mean age of participants.[13], [19], [21]-[23] Although two studies included adolescents and one included children, no additional details were provided about these participants. [13],[14] All studies enrolled a majority of male participants, ranging from 51–100% of study participants. Definitions of homelessness varied across studies and were generally inadequately described. Three studies used duration of homelessness as a criterion for study enrollment.[10], [11], [15] While duration of homelessness was provided in a total of seven studies, there were no details about participants’ moves and housing transitions. Eleven (65%) studies included information about participants’ ethnic background or country of origin. Study samples were generally comprised of a higher number of individuals from minority populations (e.g. African-American). However, no studies assessed differences in outcomes by race. Only six (35%) studies provided information about participants’ level of education, employment, or income.[10], [11], [15]-[17], [19] Health insurance coverage rates were reported in three studies in the United States ranging from 14%-47%.[10], [15], [19]

### Funding

Seven (41%) studies were supported by a government agency. Five studies reported a non-governmental source of funding and eight studies did not include a source of funding.

### Study Design and Foot Assessment

The majority of studies were observational and descriptive in nature, and only one measured the effects of a foot care intervention. Most studies involved a participant questionnaire and/or a combination of a foot assessment and retrospective chart review of health clinics that served homeless individuals. For 13 (76%) studies, foot health of homeless individuals involved a clinical assessment. Clinical foot exams were often performed by a clinician or medical student. Four (24%) studies retrieved information about foot health solely through self-reported survey data. One larger case-control study involving 698 individuals compared residents of homeless shelters with people attending a pre-travel clinic. [24] Only two studies longitudinally assessed participants’ foot concerns over time. One study compared participants who remained homeless 18 months later with participants who obtained housing. [17] One interventional study of a mobile health service examined the effect of medical treatment on diabetic foot ulcers among homeless individuals during 17.6 ± 12 months. [19] No clinical trials or qualitative studies were identified in the systematic search.

### Methodological Quality

The reported methodological quality in the majority of studies was generally moderate with a median score of 12 (range 8–20). The main study objectives were unclear in 2 (12%) studies. The main outcomes were inadequately described in 4 (24%) studies. Studies often lacked details on the sample such as mean age, sociodemographic data, recruitment procedures, and eligibility criteria. Data were often missing from reports without explanation and only 4 (24%) studies reported on participants lost to follow-up. Several studies appeared to have low internal and external validity and appeared to be insufficiently powered to detect clinically meaningful differences.

### Foot Conditions

Study participants reported a wide range of foot pathologies. Prevalence of any foot problem ranged from 9% to 65% across study populations. Calluses and corns were among the most common concerns in homeless populations, which were identified in 7.7–57% of study participants. [12], [14], [16], [19] Nail pathologies such as ingrown toenails were common, ranging from 15%-65% across study samples. [14], [16], [19] Foot infections were also highly prevalent among study populations. Tinea pedis was identified as a presenting concern in six studies. [11], [12], [14], [16], [19], [24] Rates of tinea pedis ranged from 3.2% to 38%. In addition, one case-control study involving 698 participants found that homeless individuals were significantly more likely to have tinea pedis and scratching lesions of the socks compared to people presenting to a pre-travel clinic. [24] Upon further analysis, presence of scratching lesions of the socks were independently associated with homelessness. Prevalence of pitted keratolysis of the feet (20.4%) and toenail onychomycosis (15.5%) were reported in one study. [12] Cellulitis was also found in two studies. [16], [22] Prevalence of foot injuries ranged from 24–43% in studies. [14], [19] Foot deformities (14%), trauma (6%) and fractures (2.5%) were common. [16], [21] Bunions (14%), hammertoes (7%), gout (6%), plantar warts (2–4%), and foot ulcers (0.7–4%) were also identified. [12], [14], [19] Foot problems related to neurological disorders ranged from 37–43% in one study. [14] Acute medical problems such as deep vein thrombosis, frostbite, and gangrene were also found. [16] One study found the prevalence of frostbite among homeless participants was 2%. [19] In the same study, immersion (trench) foot was observed in 5% of participants. [19]

Foot pathologies related to diabetes were found in several studies. [13], [18], [19], [23] Prevalence of diabetes ranged from 6.2–23%. Arnaud *et al*. screened 488 homeless shelter residents for diabetes which identified 35 previously diagnosed and 2 newly diagnosed individuals with diabetes. [23] They found that 41% of homeless individuals with diabetes had difficulty walking, 42% had a loss of foot sensitivity, 43% had permanently reduced mobility, and 17% had experienced lower limb amputation. One in three homeless persons with diabetes had a high or very high podiatric risk as defined by international classification, warranting regular foot care. [23] Macnee *et al*. screened 58 homeless individuals for diabetes using capillary blood sugar values and found that 12% of patients who attended the screening clinic reported foot problems. [13] In one study of homeless adults who used foot care services, 56% reported a history of diabetes, hypertension, peripheral vascular disease or a combination of the three. [18] In one study of 299 homeless shelter residents, approximately 9% self-reported having diabetes with 5% having diabetes mellitus type 2 and 4% having diabetes mellitus type 1. [19] However, 16% of participants reported numbness, 21% had tingling in their feet, and 21% had swollen feet suggesting that some individuals may have had undiagnosed diabetes. [19] In this study, 3% of participants reported having peripheral artery disease. [19]

The only interventional study identified in the review was a prospective study of 930 homeless individuals which included 21(2.3%) individuals with diabetic foot ulcers and were treated with removal of necrotic tissue, incision and drainage of infected areas and abscesses, wound care, antibiotics and analgesics as needed weekly by a mobile health services team for a mean (SD) of 17.6 ± 12 months. [25] Participants requiring complex interventions such as surgery or revascularization were hospitalized. Protective footwear was provided to all participants. Eighteen (86%) participants had significant improvement: 13 had their condition completely resolved, and 5 showed partial improvement and no longer needed medication. [25] One patient required foot amputation and negative pressure wound therapy and later died of septic shock and kidney failure. Two patients required amputation due to worsening of ulcers. [25]

One study found no significant differences in the reporting of foot problems between homeless men and women. [20] One longitudinal, non-interventional study found that homeless participants’ podiatric concerns significantly decreased over time from 12.4% at baseline to 5.7% at 18 months. [17]

#### Mobility and function

Walking was the primary means of transportation for many homeless individuals. In one study, 74% of respondents stated they were on their feet 5 hours or more each day. [19] One study found that homeless individuals walked a median of 5 miles daily. [18] Foot pain was reported by 56% of participants in one study, with 12% reporting they had pain all the time. [19] Homeless individuals were more likely to report pain in their feet when walking compared to housed participants (mean 2.0 vs. 1.7 on scale of 1–4, p = 0.003) and were more likely to report functional limitations when walking uphill (functional status score 6 vs. 8, p = 0.001). [10] However, in another study, homeless individuals were on average worried “a little” or “somewhat” about their skin, foot, or leg problems and reported associated restrictions in daily activities “a little of the time” or “not at all”. [15]

#### Foot hygiene

One study found that only 61% of homeless participants changed to a clean pair of socks daily and 72% of participants washed their feet daily. [19] In this study, 73% of participants trimmed their toenails at least once a month, and approximately 57% used lotion on their feet regularly. Seventy-six percent of participants reported keeping their feet dry, but 13% reported that they could not really feel whether their feet were dry or wet. [19] A survey conducted with 100 homeless adults examined foot hygiene attitudes and practices. [18] 52% of participants believed they needed foot care and 92% believed healthy feet were important and wanted to learn about how to maintain healthy feet. However, only 68% had access to clean water, 70% to soap, 56% to a towel, 44% to a nail clipper, 31% to a nail file, and 15% to a mirror. Only 26% reported ever having their feet examined by a health care provider. One study rated foot hygiene of participants and found that 58% had good foot hygiene, 38% had average foot hygiene with feet that were clean after simple washing, and 4% had poor foot hygiene. [21]

#### Footwear

One study found that sneakers were the most common kind of footwear among homeless individuals (84%), followed by dress shoes (28%), sandals (22%), heels (3%), boots (3%), slippers (1%), and no shoes (1%). [19] This study found that approximately 73% of participants indicated that they were able to change shoes at least every 6 months. [19]

Homeless individuals often had improperly fitting shoes. [9], [13], [22] Macnee *et al*. found that 33% of individuals who presented at a foot screening clinic had ill-fitting shoes, [13] while Schwarzkopf *et al*. found that 43.5% of homeless men had foot and shoe size mismatch of greater than 1 size and 16.9% had foot and shoe size mismatch of greater than 1.5 sizes. [9] Rates of foot and shoe size mismatch greater than 1 size were significantly higher at a free clinic serving homeless individuals compared to a diabetic foot and ankle clinic and a foot specialist private practice. [9]

#### Access to foot care

Homeless individuals were seen in shelters, general medical and foot clinics, medical centers, drop-in services, and emergency departments (EDs) for foot problems. In one study, 25% of homeless respondents reported being seen in the ED for a foot-related concern. [18] Another study found that 31% of homeless participants had visited a health professional for a foot problem. [19] Several studies reported that 20–21% of individuals who presented with a foot concern required further follow-up due to the severity of their condition. [11], [21], [22] Only one study explicitly surveyed participants about reasons for not accessing foot care services; [18] 62% of participants cited embarrassment due to the poor condition of feet, shoes, or socks as the main deterrent for receiving foot care. Among 19% of respondents who said they did not use foot care services even though they needed them, 3% said they were too busy. Other reasons included having to be around others, discomfort with others touching their feet, or not knowing where to go to receive care. When asked what would make it more likely for people to use foot care services, 27% said increased awareness. [18] One study found that independent predictors of participants who obtained foot care were longer duration of homelessness, residing in a homeless shelter, days of restricted activity, and medical comorbidities (e.g. hypertension, vision impairment, and tuberculosis). [15]

## Discussion

We systematically reviewed the published literature related to foot conditions among homeless persons. While relatively little empirical data has been published, studies report high rates of foot problems among homeless individuals across settings. Across studies included in the review, up to two thirds of homeless individuals reported a foot health concern with approximately one quarter of individuals visiting a health professional and one fifth of individuals requiring further follow-up due to the severity of their condition. [11], [18], [19], [21], [22]

Although few studies compared rates of foot problems between homeless and housed individuals, study findings suggest that homeless individuals were more likely to experience foot concerns and associated health limitations compared to housed individuals. Homeless individuals were more likely to have tinea pedis and scratching lesions of the socks compared to housed counterparts. [24] Prevalence of tinea pedis in homeless individuals across studies was similar to the prevalence in marathon runners (31%), but less than occupations such as military personnel and miners. [26] Homeless individuals were also more likely to report pain in their feet when walking compared to housed participants. Foot pain was reported in up to 56% of homeless individuals in one study, with 12% reporting constant pain, which was comparable with the prevalence of foot pain (20%) identified in a systematic review of cross-sectional studies among older adults. [27] Homeless individuals were more likely to report functional limitations with walking compared to housed individuals. [10] In one study, homeless participants who subsequently found housing were more likely to report significantly reduced podiatric concerns compared with baseline. [17]

The foot problems identified across studies represented a wide range of acute conditions and manifestations of chronic diseases. The high prevalence and severity of foot conditions can be attributed to a variety of physical, psychosocial, and service provision factors. Homeless individuals have an increased risk of physical injuries and repetitive minor trauma. [6] Poor foot hygiene, sleeping on the streets, and living in crowded environments such as homeless shelters increases exposure to pathogens and increases risk of acquiring infections. [28] Medical conditions such as frostbite, gangrene, and trench foot can occur due to lack of shelter and prolonged exposure to moist and cold environments. [6] Foot problems can also be a manifestation of chronic disease as evidenced by high rates of diabetes, peripheral vascular disease, and hypertension found across studies. [13], [18], [23]

Studies also frequently attributed foot problems in this population to poor footwear, [16], [21] and homeless individuals were more likely than their counterparts to have improperly-fitting shoes. [9], [13] Lack of access to clean socks and properly fitting shoes can cause and worsen foot problems. [6] Embarrassment related to the condition of their feet can prevent individuals from seeking appropriate health care. [13], [18] Many patients also may not be aware of foot care services or lack health insurance coverage. [10], [15] Interestingly, one study found that independent predictors of participants who obtained foot care were longer duration of homelessness, residing in a homeless shelter, days of restricted activity, and medical comorbidities. [15]

Although only one interventional study was identified in the literature review, the intervention which involved a mobile health team providing homeless individuals with medical and procedural treatment along with protective footwear for diabetic foot ulcers led to significant improvement in 86% of individuals, suggesting the effectiveness of a multifactorial treatment approach. [25]

This study adds to a growing body of literature that suggests homeless individuals experience foot problems that are often overlooked. [3], [5], [6] Given the prevalence and significant morbidity of foot health concerns, these findings have important service provision and public health implications. Homeless individuals often believe that healthy feet are important and are interested in receiving information about how to maintain adequate foot hygiene. Ensuring a private setting and cleaning individuals’ feet before the clinical exam may reduce embarrassment and encourage use of foot care services. Since these individuals may not have material and financial resources that are necessary to maintain good foot hygiene such as clean water, soap, towels, nail clippers and files, [18] service providers should ensure that individuals have access to essential foot care items. Ensuring that individuals have clean socks and properly fitting shoes could help reduce the incidence of foot problems and improve mobility. Moreover, treating foot problems in these individuals may be an important step to addressing other unmet health and social needs of this population. [29] The low rates of health insurance coverage among homeless populations included in this review suggest that health providers and health systems should explore strategies to remove barriers for this population to receive foot care. [10], [15] The findings from this review suggest that an effective interventional approach could include optimization of foot hygiene and footwear, provision of necessary medications and procedures, and addressing the social factors that predispose homeless individuals to foot problems.

### Limitations

This study was limited by a small body of literature related to foot conditions among homeless persons, with few studies specifically reporting on foot health as a primary study outcome. The review may not have been representative of the studies conducted on foot health among homeless individuals due to publication bias resulting from the small number of published articles on the topic. Low reported quality of several studies, which was evidenced by lack of details on participant characteristics and foot conditions, also limited the conclusions that could be drawn from them.

Our results are consistent with previous narrative reviews, case reports, and commentaries emphasizing the large burden of foot problems among homeless individuals, suggesting that more high quality data is needed. [3], [5], [6] Despite the prevalence of foot problems in this population, there are significant gaps between foot health needs and research studies to address these needs.

There is room for improvement in the quality of research reports given that a substantial proportion of studies did not include clear objectives and outcomes, provided limited data on participant samples, had low internal and external validity, and were insufficiently powered to detect clinically significant differences.

Future studies should assess foot health outcomes longitudinally and explore demographic and health status indicators that are associated with foot-related concerns. Comparative studies of homeless and housed individuals could yield more epidemiologic data and identify potential areas for intervention. Qualitative studies could further investigate facilitators and barriers to maintaining good foot health and accessing foot care services. Development of effective interventions to address the unmet foot health needs of this population should be prioritized. Interventional studies could explore multidisciplinary treatment approaches, provision of essential items such as clean socks and properly-fitting shoes, along with educational and outreach initiatives. Mobile interventions may be particularly effective in this population given the high burden of unmet medical and social needs.

## Conclusion

Foot conditions are very common among homeless populations. This synthesis of what is known about foot-related conditions among homeless persons has important service provision and public health implications, highlighting the need for high quality, evidence-based interventions to address the foot health needs of this population. Ultimately, targeted efforts to screen for foot problems and manage associated physical and psychosocial factors could help to improve health and social outcomes for these individuals and could potentially reduce avoidable use of costly health care services.

## Supporting Information

S1 Box Reasons for exclusion of 67 full-text articles identified from systematic literature search (DOCX)

S1 Checklist PRISMA Checklist. (DOC)
