# Supplementary material for: Foot Conditions among Homeless Persons: A Systematic Review
Source: PLoS One. 2016 Dec 9;11(12):e0167463. doi: 10.1371/journal.pone.0167463 (PMC5147925; doi:10.1371/journal.pone.0167463)
Supplement: S1 Box — (DOCX) [file pone.0167463.s001.docx]

**Foot conditions among homeless persons: a systematic review**

**Reasons for exclusion of full-text articles**

1. Schoon PM, Champlin BE, Hunt RJ. J Nurs Educ. 2012 Dec;51(12):714-8. Developing a sustainable foot care clinic in a homeless shelter within an academic-community partnership.

Reason for Exclusion: No foot health outcomes

2. Bamberg JH, Chiswell M, Toumbourou JW. Public Health Nurs. 2011 Mar-Apr;28(2):140-9. doi: 10.1111/j.1525-1446.2010.00909.x. Epub 2011 Jan 26. Use of the program explication method to explore the benefits of a service for homeless and marginalized young people.

Reason for Exclusion: No foot health outcomes

3. J Holist Nurs. 2010 Dec;28(4):244-50. doi: 10.1177/0898010110373655. Epub 2010 Jul 26.

Nightingale theory and intentional comfort touch in management of tinea pedis in vulnerable populations. Howett M, Connor A, Downes E.

Reason for Exclusion: Type of article - clinical

4. Raman SR, Jamil Z, Cosgrove J. Emerg Med J. 2011 May;28(5):450. doi: 10.1136/emj.2009.085225. Epub 2010 Apr 8. Magnetic resonance angiography unmasks frostbite injury.

Reason for Exclusion: Case report

5. Collinson S, Ward R. Br J Nurs. 2010 Jan 14-27;19(1):36-41. A nurse-led response to unmet needs of homeless migrants in inner London.

Reason for Exclusion: No foot health outcomes

6. Hedin G, Fang H. Microb Drug Resist. 2007 Winter;13(4):241-50. doi: 10.1089/mdr.2007.760.

Epidemiology of methicillin-resistant Staphylococcus aureus in Southern

Stockholm, 2000-2003.

Reason for Exclusion: Not homeless

7. Lewis SD, Peter GS, Gómez-Marín O, Bisno AL. Am J Med Sci. 2006 Dec;332(6):304-7. Risk factors for recurrent lower extremity cellulitis in a U.S. Veterans Medical Center population.

Reason for Exclusion: Not homeless

8. Feldmeier H, Kehr JD, Heukelbach J. Acta Trop. 2006 Oct;99(2-3):126-36. Epub 2006 Sep 29. A plant-based repellent protects against Tunga penetrans infestation and sand flea disease.

Reason for Exclusion: No foot health outcomes

9. Drake D. J Christ Nurs. 2005 Fall;22(4):38. Washing dirty feet.

Reason for Exclusion: Type of article - anecdotal

10. Davidson MB, Karlan VJ, Hair TL. Am J Med Qual. 2000 Jul-Aug;15(4):137-42. Effect of a pharmacist-managed diabetes care program in a free medical clinic.

Reason for Exclusion: No foot health outcomes

11. Usatine RP, Gelberg L, Smith MH, Lesser J. Am Fam Physician. 1994 Jan;49(1):139-46.

Health care for the homeless: a family medicine perspective.

Reason for Exclusion: Type of article - review

12. Packett S, Oswald N, Bronson S, Kraushar T. RN. 1991 Nov;54(11):53-5. A problem homeless patients may not mention.

Reason for Exclusion: Type of article - clinical/perspective

13. Wrenn K. Arch Intern Med. 1991 Apr;151(4):785-8. Immersion foot. A problem of the homeless in the 1990s.

Reason for Exclusion: Type of article - case report

14. Wrenn K. Ann Intern Med. 1990 Oct 15;113(8):567-9. Foot problems in homeless persons.

Reason for Exclusion: Type of article - review

15. Levoy RP. J Am Podiatry Assoc. 1974 Sep;64(9):735-6. The "runaway practice".

Reason for Exclusion: Type of article - perspective

16. Usatine R.P. Western Journal of Medicine (2000) 173:3 (160-161). The palms and soles.

Reason for Exclusion: Type of article - case report

17. Jones M. Journal of Diabetes Nursing, 2012; 16 (6): 255. [A community outreach service for vulnerable people with diabetes.](http://web.a.ebscohost.com.ezproxy.library.dal.ca/ehost/viewarticle?data=dGJyMPPp44rp2%2fdV0%2bnjisfk5Ie46bZRsKe0Sa6k63nn5Kx95uXxjL6orUmzpbBIr6qeSriqslKyqZ5oy5zyit%2fk8Xnh6ueH7N%2fiVbOotEqzr69Rrpzqeezdu33snOJ6u9m2gKTq33%2b7t8w%2b3%2bS7Sq6nr1GurrFJspzkh%2fDj34y73POE6urjkPIA&hid=4106)

Reason for Exclusion: Type of article– news/case

18. Moore A. Nursing Standard, 2013 Feb 27; 27 (26): 20-1. Anyone can be homeless.

Reason for Exclusion: Type of article – news

19. Burrett K; Podiatry Now, 2008 May; 11 (5): 49-50. [Caring for life: a student observation day.](http://web.a.ebscohost.com.ezproxy.library.dal.ca/ehost/viewarticle?data=dGJyMPPp44rp2%2fdV0%2bnjisfk5Ie46bZRsKe0Sa6k63nn5Kx95uXxjL6orUmzpbBIr6qeSriqslKyqZ5oy5zyit%2fk8Xnh6ueH7N%2fiVbOotEqzr69Rrpzqeezdu33snOJ6u9m2gKTq33%2b7t8w%2b3%2bS7Sq6mt1Gwp7FIs5zkh%2fDj34y73POE6urjkPIA&hid=4106)

Reason for Exclusion: Type of article – anecdote

20. Abraham SE; Young S; Podiatry Management, 2007 Feb; 26 (2): 135-42. [Clinical podiatry. Frostbite: diagnosis, pathophysiology, and treatment.](http://web.a.ebscohost.com.ezproxy.library.dal.ca/ehost/viewarticle?data=dGJyMPPp44rp2%2fdV0%2bnjisfk5Ie46bZRsKe0Sa6k63nn5Kx95uXxjL6orUmzpbBIr6qeSriqslKyqZ5oy5zyit%2fk8Xnh6ueH7N%2fiVbOotEqzr69Rrpzqeezdu33snOJ6u9m2gKTq33%2b7t8w%2b3%2bS7Sq6mt02wp69Pr5zkh%2fDj34y73POE6urjkPIA&hid=4106)

Reason for Exclusion: Type of article - clinical review

21. Murray S; British Journal of Nursing, 1997 Jul 10-23; 6 (13): 726, 728, 730. [Clinical. A nurse-led clinic for patients with peripheral vascular disease.](http://web.a.ebscohost.com.ezproxy.library.dal.ca/ehost/viewarticle?data=dGJyMPPp44rp2%2fdV0%2bnjisfk5Ie46bZRsKe0Sa6k63nn5Kx95uXxjL6orUmzpbBIr6qeSriqslKyqZ5oy5zyit%2fk8Xnh6ueH7N%2fiVbOotEqzr69Rrpzqeezdu33snOJ6u9m2gKTq33%2b7t8w%2b3%2bS7Sbevtkiup7RQs5zkh%2fDj34y73POE6urjkPIA&hid=4106)

Reason for Exclusion: Not homeless

22. Gilmer MJ; Australian Nursing Journal, 2008 Nov; 16 (5): 45. [Combining academia and caring for vulnerable populations.](http://web.a.ebscohost.com.ezproxy.library.dal.ca/ehost/viewarticle?data=dGJyMPPp44rp2%2fdV0%2bnjisfk5Ie46bZRsKe0Sa6k63nn5Kx95uXxjL6orUmzpbBIr6qeSriqslKyqZ5oy5zyit%2fk8Xnh6ueH7N%2fiVbOotEqzr69Rrpzqeezdu33snOJ6u9m2gKTq33%2b7t8w%2b3%2bS7Sq6nrki2rK9QtZzkh%2fDj34y73POE6urjkPIA&hid=4106)

Reason for Exclusion: Type of article - news

23. Case Management Advisor, 2013 Jun; 24 (6): 65-7. [Diabetes management takes creativity.](http://web.a.ebscohost.com.ezproxy.library.dal.ca/ehost/viewarticle?data=dGJyMPPp44rp2%2fdV0%2bnjisfk5Ie46bZRsKe0Sa6k63nn5Kx95uXxjL6orUmzpbBIr6qeSriqslKyqZ5oy5zyit%2fk8Xnh6ueH7N%2fiVbOotEqzr69Rrpzqeezdu33snOJ6u9m2gKTq33%2b7t8w%2b3%2bS7Sq6nsEmvprNIt5zkh%2fDj34y73POE6urjkPIA&hid=4106)

Reason for Exclusion: Type of article – news

24. Ries E; PT: Magazine of Physical Therapy, 2007 Dec; 15 (12): 26-9. [Enriching care to patients with low income: watchwords of cultural competence include flexibility and customization.](http://web.a.ebscohost.com.ezproxy.library.dal.ca/ehost/viewarticle?data=dGJyMPPp44rp2%2fdV0%2bnjisfk5Ie46bZRsKe0Sa6k63nn5Kx95uXxjL6orUmzpbBIr6qeSriqslKyqZ5oy5zyit%2fk8Xnh6ueH7N%2fiVbOotEqzr69Rrpzqeezdu33snOJ6u9m2gKTq33%2b7t8w%2b3%2bS7Sq6mt0%2bzprFPtZzkh%2fDj34y73POE6urjkPIA&hid=4106)

Reason for Exclusion: No foot health outcomes

25. Podiatry Management, 2011 Feb; 30 (2): 195-8. Examination.

Reason for Exclusion: Type of article – clinical quiz

26. Buchbinder, Irving; Podiatry Management, 2011 Nov-Dec; 30 (9): 73-5. [Federally Qualified Health Centers (FQHC) and Podiatry.](http://web.a.ebscohost.com.ezproxy.library.dal.ca/ehost/viewarticle?data=dGJyMPPp44rp2%2fdV0%2bnjisfk5Ie46bZRsKe0Sa6k63nn5Kx95uXxjL6orUmzpbBIr6qeSriqslKyqZ5oy5zyit%2fk8Xnh6ueH7N%2fiVbOotEqzr69Rrpzqeezdu33snOJ6u9m2gKTq33%2b7t8w%2b3%2bS7Sq6nr0u1p7dRt5zkh%2fDj34y73POE6urjkPIA&hid=4106" \o "Federally Qualified Health Centers (FQHC) and Podiatry.)

Reason for Exclusion: Type of article – perspective

27. Making the Rounds in Health, Faith & Ethics, 1995 Dec 18; 1 (8): 1. [Giving back to the community.](http://web.a.ebscohost.com.ezproxy.library.dal.ca/ehost/viewarticle?data=dGJyMPPp44rp2%2fdV0%2bnjisfk5Ie46bZRsKe0Sa6k63nn5Kx95uXxjL6orUmzpbBIr6qeSriqslKyqZ5oy5zyit%2fk8Xnh6ueH7N%2fiVbOotEqzr69Rrpzqeezdu33snOJ6u9m2gKTq33%2b7t8w%2b3%2bS7Sq6mt0y0rLJRsJzkh%2fDj34y73POE6urjkPIA&hid=4106)

Reason for Exclusion: Type of article – interview

28. Pfeil M; Howe A; Primary Health Care, 2004 Sep; 14 (7): 23-6. [Health care for hard-to-reach groups.](http://web.a.ebscohost.com.ezproxy.library.dal.ca/ehost/viewarticle?data=dGJyMPPp44rp2%2fdV0%2bnjisfk5Ie46bZRsKe0Sa6k63nn5Kx95uXxjL6orUmzpbBIr6qeSriqslKyqZ5oy5zyit%2fk8Xnh6ueH7N%2fiVbOotEqzr69Rrpzqeezdu33snOJ6u9m2gKTq33%2b7t8w%2b3%2bS7Sq6ms0ivq7FJsJzkh%2fDj34y73POE6urjkPIA&hid=4106)

Reason for Exclusion: No foot health outcomes

29. Ainsworth S; Practice Nurse, 2008 Dec 12; 36 (10): 34-5. [Helping the homeless.](http://web.a.ebscohost.com.ezproxy.library.dal.ca/ehost/viewarticle?data=dGJyMPPp44rp2%2fdV0%2bnjisfk5Ie46bZRsKe0Sa6k63nn5Kx95uXxjL6orUmzpbBIr6qeSriqslKyqZ5oy5zyit%2fk8Xnh6ueH7N%2fiVbOotEqzr69Rrpzqeezdu33snOJ6u9m2gKTq33%2b7t8w%2b3%2bS7Sq6nrkmzrK9JtJzkh%2fDj34y73POE6urjkPIA&hid=4106)

Reason for Exclusion: Type of article – news

30. Gibeau JL; Topics in Geriatric Rehabilitation, 2001 Sep; 17 (1): 22-52. [Home free: an evolving journey in eradicating elder homelessness... Committee To End Elder Homelessness (CEEH).](http://web.a.ebscohost.com.ezproxy.library.dal.ca/ehost/viewarticle?data=dGJyMPPp44rp2%2fdV0%2bnjisfk5Ie46bZRsKe0Sa6k63nn5Kx95uXxjL6orUmzpbBIr6qeSriqslKyqZ5oy5zyit%2fk8Xnh6ueH7N%2fiVbOotEqzr69Rrpzqeezdu33snOJ6u9m2gKTq33%2b7t8w%2b3%2bS7Sq6msEi0prdIsZzkh%2fDj34y73POE6urjkPIA&hid=4106)

Reason for Exclusion: No foot health outcomes

31. Hatton DC; Kleffel D; Bennett S; Gaffrey EAN; Journal of Community Health Nursing, 2001 Spring; 18 (1): 25-34. [Homeless women and children's access to health care: a paradox.](http://web.a.ebscohost.com.ezproxy.library.dal.ca/ehost/viewarticle?data=dGJyMPPp44rp2%2fdV0%2bnjisfk5Ie46bZRsKe0Sa6k63nn5Kx95uXxjL6orUmzpbBIr6qeSriqslKyqZ5oy5zyit%2fk8Xnh6ueH7N%2fiVbOotEqzr69Rrpzqeezdu33snOJ6u9m2gKTq33%2b7t8w%2b3%2bS7Sq6mr0izprFKtZzkh%2fDj34y73POE6urjkPIA&hid=4106)

Reason for Exclusion: No foot health outcomes

32. Homisak, Lynn; Podiatry Management, 2012 Aug; 31 (6): 61-3. [How Do You Handle the Disappearing Patient?](http://web.a.ebscohost.com.ezproxy.library.dal.ca/ehost/viewarticle?data=dGJyMPPp44rp2%2fdV0%2bnjisfk5Ie46bZRsKe0Sa6k63nn5Kx95uXxjL6orUmzpbBIr6qeSriqslKyqZ5oy5zyit%2fk8Xnh6ueH7N%2fiVbOotEqzr69Rrpzqeezdu33snOJ6u9m2gKTq33%2b7t8w%2b3%2bS7Sq6nr06xrq5Qt5zkh%2fDj34y73POE6urjkPIA&hid=4106)

Reason for Exclusion: Type of article – question and answer

33. Westwood C; Nursing Standard, 2007 Oct 31; 22 (8): 63. [Inner strength.](http://web.a.ebscohost.com.ezproxy.library.dal.ca/ehost/viewarticle?data=dGJyMPPp44rp2%2fdV0%2bnjisfk5Ie46bZRsKe0Sa6k63nn5Kx95uXxjL6orUmzpbBIr6qeSriqslKyqZ5oy5zyit%2fk8Xnh6ueH7N%2fiVbOotEqzr69Rrpzqeezdu33snOJ6u9m2gKTq33%2b7t8w%2b3%2bS7Sq6mt0%2byqrZMsJzkh%2fDj34y73POE6urjkPIA&hid=4106)

Reason for Exclusion: Type of article – perspective

34. Owens C; Goble R; Gray DP; Journal of Interprofessional Care, 1999 Aug; 13 (3): 277. [Involvement in multiprofessional continuing education: a local survey of 24 health care professions.](http://web.a.ebscohost.com.ezproxy.library.dal.ca/ehost/viewarticle?data=dGJyMPPp44rp2%2fdV0%2bnjisfk5Ie46bZRsKe0Sa6k63nn5Kx95uXxjL6orUmzpbBIr6qeSriqslKyqZ5oy5zyit%2fk8Xnh6ueH7N%2fiVbOotEqzr69Rrpzqeezdu33snOJ6u9m2gKTq33%2b7t8w%2b3%2bS7Sq6mt0yyrK9Ot5zkh%2fDj34y73POE6urjkPIA&hid=4106)

Reason for Exclusion: Not homeless

35. O'Toole, Thomas P.; Bourgault, Claire; Johnson, Erin E.; Redihan, Stephen G.; Borgia, Matthew; Aiello, Riccardo; Kane, Vincent; American Journal of Public Health, 2013 Dec; 103 (S2): Supplement: S374-9. [New to Care: Demands on a Health System When Homeless Veterans Are Enrolled in a Medical Home Model.](http://web.a.ebscohost.com.ezproxy.library.dal.ca/ehost/viewarticle?data=dGJyMPPp44rp2%2fdV0%2bnjisfk5Ie46bZRsKe0Sa6k63nn5Kx95uXxjL6orUmzpbBIr6qeSriqslKyqZ5oy5zyit%2fk8Xnh6ueH7N%2fiVbOotEqzr69Rrpzqeezdu33snOJ6u9m2gKTq33%2b7t8w%2b3%2bS7Sq6nsEu2p7FIs5zkh%2fDj34y73POE6urjkPIA&hid=4106)

Reason for Exclusion: No foot health outcomes

36. Connecticut Nursing News, 2011 Jun-Aug; 84 (2): 11. [Nursing outside the box. Nursing professor shows how public health works outside the classroom.](http://web.a.ebscohost.com.ezproxy.library.dal.ca/ehost/viewarticle?data=dGJyMPPp44rp2%2fdV0%2bnjisfk5Ie46bZRsKe0Sa6k63nn5Kx95uXxjL6orUmzpbBIr6qeSriqslKyqZ5oy5zyit%2fk8Xnh6ueH7N%2fiVbOotEqzr69Rrpzqeezdu33snOJ6u9m2gKTq33%2b7t8w%2b3%2bS7Sq6nr0m3rrBLs5zkh%2fDj34y73POE6urjkPIA&hid=4106)

Reason for Exclusion: Type of article – news

37. Butler-Ajibade, Phoebe; Booth, William; Burwell, Cynthia; ABNF Journal, 2012 Spring; 23 (2): 34-7. [Partnering with the Black Church: Recipe for Promoting Heart Health in the Stroke Belt.](http://web.a.ebscohost.com.ezproxy.library.dal.ca/ehost/viewarticle?data=dGJyMPPp44rp2%2fdV0%2bnjisfk5Ie46bZRsKe0Sa6k63nn5Kx95uXxjL6orUmzpbBIr6qeSriqslKyqZ5oy5zyit%2fk8Xnh6ueH7N%2fiVbOotEqzr69Rrpzqeezdu33snOJ6u9m2gKTq33%2b7t8w%2b3%2bS7Sq6nr02zqLZKr5zkh%2fDj34y73POE6urjkPIA&hid=4106)

Type of article - perspective

38. ACCNS Journal for Community Nurses, 2005 Apr; 10 (1): 9-11. [Praxis -- research and issues in community nursing. ACCNS Colloquium 2004 Brisbane -- theme: nursing in different communities.](http://web.a.ebscohost.com.ezproxy.library.dal.ca/ehost/viewarticle?data=dGJyMPPp44rp2%2fdV0%2bnjisfk5Ie46bZRsKe0Sa6k63nn5Kx95uXxjL6orUmzpbBIr6qeSriqslKyqZ5oy5zyit%2fk8Xnh6ueH7N%2fiVbOotEqzr69Rrpzqeezdu33snOJ6u9m2gKTq33%2b7t8w%2b3%2bS7Sq6ms0muqbdLr5zkh%2fDj34y73POE6urjkPIA&hid=4106)

Reason for Exclusion: No foot health outcomes

39. Muñoz JP; Reichenbach D; Hansen AMW; Work, 2005; 25 (3): 241-52. [Project Employ: engineering hope and breaking down barriers to homelessness.](http://web.a.ebscohost.com.ezproxy.library.dal.ca/ehost/viewarticle?data=dGJyMPPp44rp2%2fdV0%2bnjisfk5Ie46bZRsKe0Sa6k63nn5Kx95uXxjL6orUmzpbBIr6qeSriqslKyqZ5oy5zyit%2fk8Xnh6ueH7N%2fiVbOotEqzr69Rrpzqeezdu33snOJ6u9m2gKTq33%2b7t8w%2b3%2bS7Sq6mt0i1qq9RsZzkh%2fDj34y73POE6urjkPIA&hid=4106)

Reason for Exclusion: No foot health outcomes

40. Luck J; Andersen R; Wenzel S; Arangua L; Wood D; Gelberg L; Journal of Ambulatory Care Management, 2002 Apr; 25 (2): 53-67. [Providers of primary care to homeless women in Los Angeles County.](http://web.a.ebscohost.com.ezproxy.library.dal.ca/ehost/viewarticle?data=dGJyMPPp44rp2%2fdV0%2bnjisfk5Ie46bZRsKe0Sa6k63nn5Kx95uXxjL6orUmzpbBIr6qeSriqslKyqZ5oy5zyit%2fk8Xnh6ueH7N%2fiVbOotEqzr69Rrpzqeezdu33snOJ6u9m2gKTq33%2b7t8w%2b3%2bS7Sq6msEi0rLdPrpzkh%2fDj34y73POE6urjkPIA&hid=4106)

Reason for Exclusion: No foot health outcomes

41. Vesely R; Modern Healthcare, 2010 Mar 15; 40 (11): 6-7, 16. [The root of the problem: with Cover the Uninsured Week under way, Carolinas battle with access, preventable ER visits---even of the dental variety.](http://web.a.ebscohost.com.ezproxy.library.dal.ca/ehost/viewarticle?data=dGJyMPPp44rp2%2fdV0%2bnjisfk5Ie46bZRsKe0Sa6k63nn5Kx95uXxjL6orUmzpbBIr6qeSriqslKyqZ5oy5zyit%2fk8Xnh6ueH7N%2fiVbOotEqzr69Rrpzqeezdu33snOJ6u9m2gKTq33%2b7t8w%2b3%2bS7Sq6nrk22rLRKtpzkh%2fDj34y73POE6urjkPIA&hid=4106)

Reason for Exclusion: Type of article – news

42. Porche DJ; Journal for Nurse Practitioners, 2006 Mar; 2 (3): 152-3. [Tinea pedis: a common male foot problem.](http://web.a.ebscohost.com.ezproxy.library.dal.ca/ehost/viewarticle?data=dGJyMPPp44rp2%2fdV0%2bnjisfk5Ie46bZRsKe0Sa6k63nn5Kx95uXxjL6orUmzpbBIr6qeSriqslKyqZ5oy5zyit%2fk8Xnh6ueH7N%2fiVbOotEqzr69Rrpzqeezdu33snOJ6u9m2gKTq33%2b7t8w%2b3%2bS7Sq6mt0myqLNJtZzkh%2fDj34y73POE6urjkPIA&hid=4106)

Reason for Exclusion: Type of article – clinical review

43. Pickersgill, Frances; Nursing Standard, 2011 Nov 23-29; 26 (12): 61. [Try and unconventional shift.](http://web.a.ebscohost.com.ezproxy.library.dal.ca/ehost/viewarticle?data=dGJyMPPp44rp2%2fdV0%2bnjisfk5Ie46bZRsKe0Sa6k63nn5Kx95uXxjL6orUmzpbBIr6qeSriqslKyqZ5oy5zyit%2fk8Xnh6ueH7N%2fiVbOotEqzr69Rrpzqeezdu33snOJ6u9m2gKTq33%2b7t8w%2b3%2bS7Sq6nr0u2rLdRrpzkh%2fDj34y73POE6urjkPIA&hid=4106)

Reason for Exclusion: Type of article – news

44. Blum, Don R.; Podiatry Management, Aug2015; 34(6): 28-28. [Clinic Conundrum.](http://web.a.ebscohost.com.ezproxy.library.dal.ca/ehost/viewarticle/render?data=dGJyMPPp44rp2%2fdV0%2bnjisfk5Ie46bZRsKe0Sa6k63nn5Kx95uXxjL6trUm0pbBIr6yeSrirtFKuq55oy5zyit%2fk8Xnh6ueH7N%2fiVaust0quqrFMta2khN%2fk5VXj5KR84LPhUOac8nnls79mpNfsVa%2bmt1CxprFJspzkh%2fDj34y73POE6urjkPIA&vid=14&sid=0183e125-2dd5-4e5f-b7cb-c338c4a2dca1@sessionmgr4002&hid=4114)

Reason for Exclusion: Not homeless

45. Samimi, Roody; Dale, Robert; Kiel, Brian; Podiatry Management, Aug2015; 34(6): 26-28. [Diabetic Ulcer.](http://web.a.ebscohost.com.ezproxy.library.dal.ca/ehost/viewarticle/render?data=dGJyMPPp44rp2%2fdV0%2bnjisfk5Ie46bZRsKe0Sa6k63nn5Kx95uXxjL6trUm0pbBIr6yeSrirtFKuq55oy5zyit%2fk8Xnh6ueH7N%2fiVaust0quqrFMta2khN%2fk5VXj5KR84LPhUOac8nnls79mpNfsVa%2bmt1CxprFJr5zkh%2fDj34y73POE6urjkPIA&vid=14&sid=0183e125-2dd5-4e5f-b7cb-c338c4a2dca1@sessionmgr4002&hid=4114)

Reason for Exclusion: Type of article – letter

46. Carver, Edward W.; Posa, Raymond F.; Podiatry Management, Aug2015; 34(6): 26-26. [Holding Us Hostage?](http://web.a.ebscohost.com.ezproxy.library.dal.ca/ehost/viewarticle/render?data=dGJyMPPp44rp2%2fdV0%2bnjisfk5Ie46bZRsKe0Sa6k63nn5Kx95uXxjL6trUm0pbBIr6yeSrirtFKuq55oy5zyit%2fk8Xnh6ueH7N%2fiVaust0quqrFMta2khN%2fk5VXj5KR84LPhUOac8nnls79mpNfsVa%2bmt1CxprFIt5zkh%2fDj34y73POE6urjkPIA&vid=14&sid=0183e125-2dd5-4e5f-b7cb-c338c4a2dca1@sessionmgr4002&hid=4114)

Reason for Exclusion: Not homeless

47. Nursing Standard, 10/15/2014; 29(7): 10-10. [IN BRIEF.](http://web.a.ebscohost.com.ezproxy.library.dal.ca/ehost/viewarticle/render?data=dGJyMPPp44rp2%2fdV0%2bnjisfk5Ie46bZRsKe0Sa6k63nn5Kx95uXxjL6trUm0pbBIr6yeSrirtFKuq55oy5zyit%2fk8Xnh6ueH7N%2fiVaust0quqrFMta2khN%2fk5VXj5KR84LPhUOac8nnls79mpNfsVa%2bmsVGuq7RPt5zkh%2fDj34y73POE6urjkPIA&vid=14&sid=0183e125-2dd5-4e5f-b7cb-c338c4a2dca1@sessionmgr4002&hid=4114)

Reason for Exclusion: Type of article – news

48. Chen, H Carrie; Sheu, Leslie; O'Sullivan, Patricia; Cate, Olle; Teherani, Arianne; Medical Education, Feb2014; 48(2): 136-145. [Legitimate workplace roles and activities for early learners.](http://web.a.ebscohost.com.ezproxy.library.dal.ca/ehost/viewarticle/render?data=dGJyMPPp44rp2%2fdV0%2bnjisfk5Ie46bZRsKe0Sa6k63nn5Kx95uXxjL6trUm0pbBIr6yeSrirtFKuq55oy5zyit%2fk8Xnh6ueH7N%2fiVaust0quqrFMta2khN%2fk5VXj5KR84LPhUOac8nnls79mpNfsVa%2bmskmxqbFJspzkh%2fDj34y73POE6urjkPIA&vid=14&sid=0183e125-2dd5-4e5f-b7cb-c338c4a2dca1@sessionmgr4002&hid=4114)

Reason for Exclusion: No foot health outcomes

49. Smith, Gary S.; Christina, Jim; Podiatry Management, Aug2015; 34(6): 26-26. [Medicare Penalties.](http://web.a.ebscohost.com.ezproxy.library.dal.ca/ehost/viewarticle/render?data=dGJyMPPp44rp2%2fdV0%2bnjisfk5Ie46bZRsKe0Sa6k63nn5Kx95uXxjL6trUm0pbBIr6yeSrirtFKuq55oy5zyit%2fk8Xnh6ueH7N%2fiVaust0quqrFMta2khN%2fk5VXj5KR84LPhUOac8nnls79mpNfsVa%2bmt1CxprFItpzkh%2fDj34y73POE6urjkPIA&vid=14&sid=0183e125-2dd5-4e5f-b7cb-c338c4a2dca1@sessionmgr4002&hid=4114)

Reason for Exclusion: Not homeless

50. Poggio, Tony; Podiatry Management, Aug2015; 34(6): 28-28. New Codes.

Reason for Exclusion: Not homeless

51. Ribotsky, Bret; Podiatry Management, Aug2015; 34(6): 28-28. [Newest Ripoff.](http://web.a.ebscohost.com.ezproxy.library.dal.ca/ehost/viewarticle/render?data=dGJyMPPp44rp2%2fdV0%2bnjisfk5Ie46bZRsKe0Sa6k63nn5Kx95uXxjL6trUm0pbBIr6yeSrirtFKuq55oy5zyit%2fk8Xnh6ueH7N%2fiVaust0quqrFMta2khN%2fk5VXj5KR84LPhUOac8nnls79mpNfsVa%2bmt1CxprFJsZzkh%2fDj34y73POE6urjkPIA&vid=14&sid=0183e125-2dd5-4e5f-b7cb-c338c4a2dca1@sessionmgr4002&hid=4114)

Reason for Exclusion: Not homeless

52. Sheridan, Hugh; Kaniadakis, Steven J.; Podiatry Management, Aug2015; 34(6): 26-26. [PM News Benchmark.](http://web.a.ebscohost.com.ezproxy.library.dal.ca/ehost/viewarticle/render?data=dGJyMPPp44rp2%2fdV0%2bnjisfk5Ie46bZRsKe0Sa6k63nn5Kx95uXxjL6trUm0pbBIr6yeSrirtFKuq55oy5zyit%2fk8Xnh6ueH7N%2fiVaust0quqrFMta2khN%2fk5VXj5KR84LPhUOac8nnls79mpNfsVa%2bmt1CxprFJrpzkh%2fDj34y73POE6urjkPIA&vid=14&sid=0183e125-2dd5-4e5f-b7cb-c338c4a2dca1@sessionmgr4002&hid=4114)

Reason for Exclusion: Not homeless

53. Podiatry Review, Mar/Apr2015; 72(2): 30-30. [Podiatrist knowledge needed to develop new resource for working with the homeless.](http://web.a.ebscohost.com.ezproxy.library.dal.ca/ehost/viewarticle/render?data=dGJyMPPp44rp2%2fdV0%2bnjisfk5Ie46bZRsKe0Sa6k63nn5Kx95uXxjL6trUm0pbBIr6yeSrirtFKuq55oy5zyit%2fk8Xnh6ueH7N%2fiVaust0quqrFMta2khN%2fk5VXj5KR84LPhUOac8nnls79mpNfsVa%2bmtU%2b1rLZOrpzkh%2fDj34y73POE6urjkPIA&vid=14&sid=0183e125-2dd5-4e5f-b7cb-c338c4a2dca1@sessionmgr4002&hid=4114)

Reason for Exclusion: Type of article – news

54. Chaskin, Dan; Podiatry Management, Aug2015; 34(6): 24-26. [Shredding Query.](http://web.a.ebscohost.com.ezproxy.library.dal.ca/ehost/viewarticle/render?data=dGJyMPPp44rp2%2fdV0%2bnjisfk5Ie46bZRsKe0Sa6k63nn5Kx95uXxjL6trUm0pbBIr6yeSrirtFKuq55oy5zyit%2fk8Xnh6ueH7N%2fiVaust0quqrFMta2khN%2fk5VXj5KR84LPhUOac8nnls79mpNfsVa%2bmt1CxprFItZzkh%2fDj34y73POE6urjkPIA&vid=14&sid=0183e125-2dd5-4e5f-b7cb-c338c4a2dca1@sessionmgr4002&hid=4114)

Reason for Exclusion: Not homeless

55. Scantlebury, Linda; Podiatry Review, Mar/Apr2015; 72(2): 26-27. [Volunteering with Crisis.](http://web.a.ebscohost.com.ezproxy.library.dal.ca/ehost/viewarticle/render?data=dGJyMPPp44rp2%2fdV0%2bnjisfk5Ie46bZRsKe0Sa6k63nn5Kx95uXxjL6trUm0pbBIr6yeSrirtFKuq55oy5zyit%2fk8Xnh6ueH7N%2fiVaust0quqrFMta2khN%2fk5VXj5KR84LPhUOac8nnls79mpNfsVa%2bmtU%2b1rLZNtZzkh%2fDj34y73POE6urjkPIA&vid=14&sid=0183e125-2dd5-4e5f-b7cb-c338c4a2dca1@sessionmgr4002&hid=4114)

Reason for Exclusion: Type of article – anecdotal

56. Curran K(1), Drust B(2), Murphy R(2), Pringle A(3), Richardson D(2). Public Health. 2016 Jun;135:14-22. doi: 10.1016/j.puhe.2016.02.008. Epub 2016 Apr 20. The challenge and impact of engaging hard-to-reach populations in regular physical activity and health behaviours: an examination of an English Premier League 'Football in the Community' men's health programme.

Reason for Exclusion: No foot health outcomes

57. Nurs Stand. 2015 Oct 7;30(6):16. doi: 10.7748/ns.30.6.16.s20. Foot health and homelessness.

Reason for Exclusion: Type of article – clinical article

58. Anderson SG, Narayanan RP, Malipatil NS, Roberts H, Dunn G, Heald AH. Exp Clin Endocrinol Diabetes. 2015 Jul;123(7):423-7. doi: 10.1055/s-0035-1549966. Epub 2015 Jun 11. Socioeconomic deprivation independently predicts painful diabetic neuropathy in type 2 diabetes.

Reason for Exclusion: Not homeless

59. Haddad MB, Foote MK, Ray SM, Maggio DM, Sales RM, Kim MJ, Kempker RR, Spaulding AC. Open Forum Infect Dis. 2014 Jun 30;1(1):ofu041. doi: 10.1093/ofid/ofu041.

eCollection 2014. Substantial overlap between incarceration and tuberculosis in atlanta, georgia,

2011.

Reason for Exclusion: No foot health outcomes

60. Helge EW(1), Randers MB, Hornstrup T, Nielsen JJ, Blackwell J, Jackman SR,

Krustrup P. Scand J Med Sci Sports. 2014 Aug;24 Suppl 1:122-9. doi: 10.1111/sms.12244.

Street football is a feasible health-enhancing activity for homeless men: biochemical bone marker profile and balance improved.

Reason for Exclusion: No foot health outcomes

61. Nguyen S. Kohn J. Tsu L. Buckley K. Pharmacotherapy (2014) 34:10 (e273). October 2014. Evaluation of pharmacist-provided women's health education seminars in the underserved female population

Reason for Exclusion: Type of article - abstract

62. Rajagopal A. Mintz E. Reese L. Chest (2014) 146:4 MEETING ABSTRACT. October 2014

Daptomycin-induced eosinophilic pneumonia without peripheral eosinophilia

Reason for Exclusion: No foot health outcomes

63. Stevens L. Goode J. Gatewood S. Moczygemba L. Journal of the American Pharmacists Association (2014) 54:2 (e105). March-April 2014. Project impact diabetes: Outcomes of diabetes awareness to reach excellence-a difference in the homeless

Reason for Exclusion: Type of article - abstract

64. Gazin P. Brouqui P. Antibiotiques (2004) 6:3 (175-179). Sep 2004. Infectious diseases in homeless persons.

Reason for Exclusion: Type of article – not original research

65. van Laere IR, Buster MC. Ned Tijdschr Geneeskd. 2001 Jun 16;145(24):1156-60. Health problems of homeless people attending the outreach primary care surgeries in Amsterdam.

Reason for Exclusion: No clear foot health outcomes

66. Bouffard L, Hébert F, Lemieux MJ, Perron G, Tremblay L. Infirm Que. 2000 Nov-Dec;8(2):40-1. Migrant patients and foot care.

Reason for Exclusion: Type of article - Not original research

67. Pessel-Nobrega M, Raymond F. Soins. 2010 Apr;(744 Suppl):S4, S6-8. Precarious state of diabetes.

Reason for Exclusion: Type of article - Not original research
